# Discovering potential interactions between rare diseases and COVID-19 by combining mechanistic models of viral infection with statistical modeling

**DOI:** 10.1093/hmg/ddac007

**Published:** 2022-01-12

**Authors:** Macarena López-Sánchez, Carlos Loucera, María Peña-Chilet, Joaquín Dopazo

**Affiliations:** Clinical Bioinformatics Area, Fundación Progreso y Salud (FPS), CDCA, Hospital Virgen del Rocio, Sevilla 41013, Spain; Computational Systems Medicine, Institute of Biomedicine of Seville (IBIS), Hospital Virgen del Rocio, Sevilla 41013, Spain; Clinical Bioinformatics Area, Fundación Progreso y Salud (FPS), CDCA, Hospital Virgen del Rocio, Sevilla 41013, Spain; Computational Systems Medicine, Institute of Biomedicine of Seville (IBIS), Hospital Virgen del Rocio, Sevilla 41013, Spain; Clinical Bioinformatics Area, Fundación Progreso y Salud (FPS), CDCA, Hospital Virgen del Rocio, Sevilla 41013, Spain; Computational Systems Medicine, Institute of Biomedicine of Seville (IBIS), Hospital Virgen del Rocio, Sevilla 41013, Spain; Bioinformatics in Rare Diseases (BiER), Centro de Investigación Biomédica en Red de Enfermedades Raras (CIBERER), FPS, Hospital Virgen del Rocío, Sevilla 41013, Spain; Clinical Bioinformatics Area, Fundación Progreso y Salud (FPS), CDCA, Hospital Virgen del Rocio, Sevilla 41013, Spain; Computational Systems Medicine, Institute of Biomedicine of Seville (IBIS), Hospital Virgen del Rocio, Sevilla 41013, Spain; Bioinformatics in Rare Diseases (BiER), Centro de Investigación Biomédica en Red de Enfermedades Raras (CIBERER), FPS, Hospital Virgen del Rocío, Sevilla 41013, Spain; FPS/ELIXIR-es, Hospital Virgen del Rocío, Sevilla 42013, Spain

## Abstract

Recent studies have demonstrated a relevant role of the host genetics in the coronavirus disease 2019 (COVID-19) prognosis. Most of the 7000 rare diseases described to date have a genetic component, typically highly penetrant. However, this vast spectrum of genetic variability remains yet unexplored with respect to possible interactions with COVID-19. Here, a mathematical mechanistic model of the COVID-19 molecular disease mechanism has been used to detect potential interactions between rare disease genes and the COVID-19 infection process and downstream consequences. Out of the 2518 disease genes analyzed, causative of 3854 rare diseases, a total of 254 genes have a direct effect on the COVID-19 molecular disease mechanism and 207 have an indirect effect revealed by a significant strong correlation. This remarkable potential of interaction occurs for >300 rare diseases. Mechanistic modeling of COVID-19 disease map has allowed a holistic systematic analysis of the potential interactions between the loss of function in known rare disease genes and the pathological consequences of COVID-19 infection. The results identify links between disease genes and COVID-19 hallmarks and demonstrate the usefulness of the proposed approach for future preventive measures in some rare diseases.

## Introduction

Although rare diseases affect a small number of patients [fewer than 1 in each 2000 people in the European Union and similar proportions in other countries (1)], there are >7000 of them already described (2). This results in a cumulated prevalence of 5–8% of the population (3), equivalent to those observed in many common diseases. It has been estimated that >80% of rare diseases have a genetic etiology (3), which suggests that a large number of genes must be involved in these pathologies. In fact, >7000 gene-disease associations have been described to date and it has been estimated that each year 300 new gene-disease associations are reported to the online mendelian inheritance in man (OMIM) database (4). The most common effect of these mutations is a total or partial loss of function (LoF), being this feature one of most widely used criteria for variant prioritization in the search for new disease genes (5,6). Therefore, many mutations causing rare diseases can be considered natural knock-outs (or knock-downs) and, consequently, some of them might have downstream consequences on other biological processes of the cell beyond the ones that cause the most prominent phenotype of the disease. Since rare disease phenotypes tend to be highly penetrant, with severe pathological consequences, secondary effects produced by the downstream consequences of the causal mutations are often considered comparatively minor problems and tend to be ignored. However, given the large number of genes involved in the whole set of rare diseases and the complex network of interaction among genes in the cell (7,8), there must be few cellular processes that remain unaffected by at least one rare disease (9).

Similarly, viruses hijack and indirectly affect many different biological processes during their infection cycle. In fact, interactions between virus infections and diseases are more than likely, and not necessarily negative for the patient, as demonstrated by a recently described remission of Hodgkin lymphoma induced by the severe acute respiratory syndrome coronavirus 2 (SARS-CoV-2) viral infection (10). Moreover, variants in specific human genes are known to have a determinant effect on the severity of coronavirus disease 2019 (COVID-19) infection (11,12). In the case of rare diseases, the likelihood of overlap between biological processes affected by rare disease mutations and viral infections is not negligible. Specifically, in the case of SARS-CoV-2, the molecular mechanism of infection is currently quite well known, considering that it is a recent emergent virus (13–15). Despite this potential range of functional connections between rare diseases and COVID-19, the cases reported to date are anecdotic, like the Kawasaki-like complication (16) with serious consequences for patients such as delays or even missed diagnosis (17), evidencing that the interactions between rare diseases and COVID-19 remains largely unexplored.

The exhaustive activity of the scientific community to fight the COVID-19 pandemics allowed the rapid generation of detailed knowledge on the COVID-19 disease mechanism. SARS-CoV-2 infection affects human molecular pathways directly involved in the viral infection, as well as other ones downstream that account for symptoms such as enhanced immune response ending in fatal cytokine storms (18), disseminated intravascular coagulopathies (19), generalized systemic inflammatory response syndromes that can lead to septic shock (20) and even lymphocyte exhaustion (21). All this knowledge defines a well-curated set of biological pathways known as COVID-19 disease map (13).

The COVID-19 disease map can be used to define a mathematical model that provides a mechanistic approach to the functional activity of the cell that ultimately accounts for the phenotype of the diseased individual. These mechanistic models can quantify the intensity of signal transduction from transcriptomic measurements, and consequently can quantify the activity of the different signaling circuits and ultimately the functions that these triggers within the cell. Since they convey the notion of causality these models can also be used to simulate the effects of perturbations like gene LoFs associated with rare diseases.

Mechanistic models have successfully been used to understand disease mechanisms in cancers (22–25), rare (26) and common (27) diseases as well as to reveal mechanisms of action of drugs (28). Of particular interest for the study of the effect of rare disease genes over the COVID-19 disease mechanism is illustrated by a recently published drug repurposing study, in which mechanistic models were used to find causal activities of proteins (targets for drugs indicated for other diseases) over the specific signaling circuits involved in the COVID-19 disease (29).

Therefore, the large number of genes causative of rare disease genes along with the many human pathways affected by the infection suggest that the likelihood of potential interactions between a number of rare diseases and the SARS-CoV-2 infection process and their downstream pathologic consequences is not negligible. Here, a mechanistic model of the recently produced COVID-19 disease map (13,14) will be used to find rare disease genes whose LoF would have an effect over the different functional hallmarks of the coronavirus infection. This innovative strategy has allowed to detect, among the >7000 rare diseases described, a total of 365 that have a potential direct interaction with the COVID-19 molecular mechanism of infection and downstream consequences, 255 diseases with potential indirect interaction and 69 diseases with both direct and indirect. This figure represents ~10% of the total number of rare diseases described, which suggests that the extent of potential interactions between rare and infectious diseases is not negligible. The approaches presented here can help to preventively detect rare diseases for which special care should be taken in the event of infections by specific pathogens, like the case of SARS-CoV-2 described in this work.

## Results

### Causal effect of LoF of rare disease genes integral to the COVID-19 disease map circuits

A total of 2518 genes reported to be causative of a total of 3854 rare diseases were described in Orphanet repository (see Supplementary Material, Table S1). Among these, 359 genes were located within signaling circuits belonging to the COVID-19 disease map (13,14), being part of a total of 270 circuits. Table 1 shows the number of rare diseases, clustered by rare disease type, that have been found to interact with the COVID-19.

**Table 1:** Number of rare diseases with a direct or indirect (or both) potential effect over the COVID-19 molecular mechanism, groped by Orphanet disease categories

Rare disease type	Direct	Indirect	Both
Developmental defect during embryogenesis	88	61	19
Neurologic	37	75	7
Immune	40	16	18
Neoplastic	51	9	10
Bone	33	14	3
Endocrine	26	7	2
Skin	17	16	1
Inborn errors of metabolism	11	20	1
Systemic or rheumatologic	19	8	3
Hematologic	12	6	2
Gastroenterologic	9	4	3
Ophthalmic	5	6	0
Renal	5	3	0
Cardiac	4	2	0
Respiratory	3	0	0
Otorhinolaryngologic	0	3	0
Others	2	1	0
Rare infertility	1	1	0
Rare odontologic disease	1	0	0
Rare infectious disease	0	1	0
Rare genetic disease	0	1	0
Rare gynecologic or obstetric disease	1	0	0
Rare circulatory system disease	0	1	0
**Total**	**365**	**255**	**69**

Mechanistic models were applied to predict the effect that knock-downs of disease genes belonging to the signaling circuits have over the resulting signaling activities of the signaling circuits of the COVID-19 disease map, using the strategy described in Materials and Methods. First, gene expression data corresponding to healthy tissues were downloaded from the genotype-tissue expression (GTEx) Portal, normalized using TMM and used to estimate signaling circuit activity profiles in the COVID-19 disease map of a cohort of healthy individuals. Subsequently, knock-downs were simulated to produce a simulated cohort of affected individuals. Finally, both cohorts were compared with detect signaling circuits differentially activated because of the knock-down of the disease gene.

**Figure 1:**
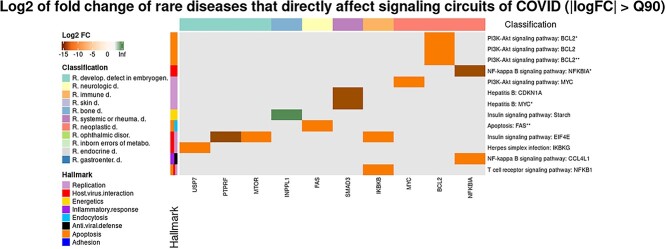
Most significant direct effects of rare disease genes over signaling circuits involved in the COVID-19. Heatmap with rare disease genes (*X* axis) present in signaling circuits (*Y* axis) of the COVID-19 disease map whose simulated knock-down produces a significant effect in the corresponding circuit. Color scale represents the log2-fold-change in the circuit when knock-downs are simulated in the mechanistic model. The upper part of the figure displays a color code representing the type of disease the gene belongs to. The left part of the figure displays a color code representing the COVID-19 hallmarks triggered by the affected circuits.

**Figure 2:**
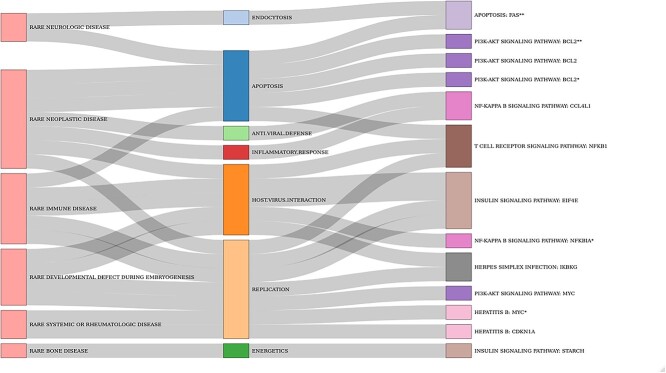
Sankey diagram of the most significant direct effects of disease genes over signaling circuits involved in the COVID 19. Sankey diagram with a summary of the connections between rare disease types with signaling circuits affected by them through COVID-19 hallmarks. The connections are obtained only for the highest 10% of the significant log2-fold-changes produced by the simulated knock-downs in the mechanistic model.

After carrying out the simulated knock-downs in the disease genes, a considerable impact over the COVID-19 disease map could be documented. A total of 254 genes, corresponding to 445 diseases, produced a significant [false discovery rate (FDR)-adjusted *P*-value < 0.05] change in the activity of a total of 264 circuits (Supplementary Material, Table S2). Supplementary Material, Figure S1 shows a summary of the significant impacts and Figure 1 represents a heatmap with the top disease genes whose knock-down cause the 10% most remarkable effect on the COVID-19 disease map (defined as | log_2_(fold-change) | > Q90). It is interesting to note that the main potential effects of natural knock-downs due to rare disease genes occur over circuits belonging to the *PI3k-Atk*, the *NF-kappa B*, *T cell receptor, Insulin* and *Apoptosis* pathways, along with other pathways related to infectious diseases like *Hepatitis B* and *Herpes simplex infection* pathways. Such signaling circuits are mainly related to replication and host-virus interaction and apoptosis, and to a lesser extent, antiviral defense, inflammatory response, energetics and endocytosis (see Fig. 2). *Rare neoplastic* and *Rare immune diseases* act pervasively through many of the COVID-19 hallmarks. Contrarily, *Rare systemic or rheumatologic diseases* seem to affect only the replication hallmark or *Rare developmental defect during embryogenesis* seem to affect mainly replication but also host-virus interaction signaling circuits. Interestingly, *Rare bone disease* acts over the energetics hallmark through the *Insulin Signaling pathway*. A more detailed diagram with all the significant effects described in Supplementary Material, Table S2 can be found in Supplementary Material, Figure S2, where other pathways, like *HIF-1, Focal adhesion, Chemoquine signaling pathways* and, especially, *Complement and coagulation cascades*, in addition to specific signaling circuits involved in the infection of other viruses, like *HTLV-1* or *Epstein–Barr*, seem to play an important role in the potential interactions between rare diseases and the COVID-19 disease map.

### Rare disease genes with potentially long-distance effect over COVID-19 disease map circuits

For disease genes laying outside the COVID-19 disease map a different approach, which utilizes a correlation-based test, has been used. The correlation of 2456 genes, that cause a total of 3782 diseases, with 276 circuits of the COVID-19 disease map across the 17 382 samples of GTEx were calculated, which involve a total of 673 723 correlations between circuits and genes external to them (genes that are integral part of the circuits were analyzed in the previous section).

**Figure 3:**
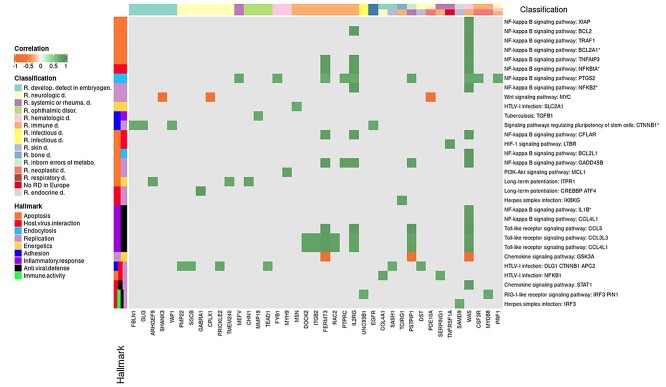
Most relevant indirect effects of disease genes over signaling circuits involved in the COVID-19. Heatmap with rare disease genes outside signaling circuits of the COVID-19 disease map with a significant high correlation (>0.75 or <−0.75) with the circuit.

**Figure 4:**
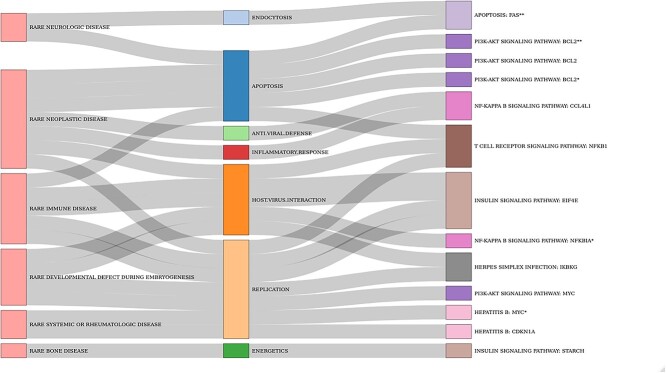
Sankey diagram of most relevant indirect effects of disease genes over signaling circuits involved in the COVID 19. Sankey diagram connecting rare disease types with signaling circuits affected by them through COVID-19 hallmarks. The connections are obtained only for the highest (> 0.75 or <−0.75) significant correlations.

After applying the correlation-based test, a total of 207 genes corresponding to 328 diseases (see Table 1) showed a significant correlation with the behavior of 63 signaling circuits of the COVID-19 disease map (Supplementary Material, Table S3). Supplementary Material, Figure S3 depicts the genes with a significant (FDR-adjusted *P*-value < 0.05) correlation with different COVID-19 disease map signaling circuits. Some circuits display a high number of interactions, like *TGF−beta signaling pathway: RPS6KB1*, which is affected by 32 genes or the *HTLV-I infection:DLG1-CTNNB1-APC2* is affected by 18 genes, in both cases causative of a broad range of diseases such as developmental defects in the embryogenesis, inborn defects of metabolism, neurologic, skin and bone, diseases and others. Supplementary Material, Figure S4 depicts the relationships between genes and circuits correlated through the corresponding hallmarks. Figure 3 presents the most highly correlated genes (|r| > 0.75) among all the significant correlations found. It is interesting to note that most of the correlations are positive, meaning that the existence of the disease as a LoF in the gene, providing that the relationship is causal, would potentially inhibit the corresponding circuit. There are a few exceptions: the *Chemokine signaling pathway:GSK3A* and the *Wnt signaling pathway:MYC* circuits, which display negative correlations with several disease genes. Like in the case of direct interactions, a considerable number of circuits belonging to the *NF-kappa B signaling pathway* displayed a significantly high correlation with disease genes. *HIF-1* and *Chemokine signaling pathways* are also among the most significantly affected pathways, although other ones, such as *WNT, Toll-like* and *RIG-I-like receptor pathways* are also affected. Again, *Rare immune disease* affects many circuits related to almost all the COVID-19 hallmarks. Similar effect is observed from *Rare hematologic disease*. Regarding the hallmarks, *Replication, host-virus interaction, apoptosis, antiviral defense* and *inflammatory response* are the most affected ones (see Fig. 4).

Although modeling LoF in disease genes as simulated knock-downs within signaling circuits of the COVID-19 disease map provides a causal explanation of their effects, the statistical modeling used for external genes provides only a significant strong correlation, which is indicative of a likely causal relation but not a proof. Therefore, a further protein–protein interaction analysis was carried out on the significant associations found to search for extra biological support that could explain the links between external genes and signaling circuit activities in the COVID-19 disease map discovered (listed in Supplementary Material, Table S3). Two thirds of the external disease genes presented a level of protein interaction with genes in the COVID-19 disease map with a confidence level from medium to high according to the STRING confidence scale (23% medium, 8.7% high and 36.5% highest). This supports the existence of a connection through protein–protein interaction that would explain the connection between the genes and the activity of the COVID-19 disease map signaling circuits. For the remainder third it may happen that the correlation was not causal or that it is not mediated by protein interactions.

Interestingly, when the most sophisticated tool, ToppGene, was applied, all the disease genes detected by the approach presented here (listed Supplementary Material, Table S3) resulted to display a significant connection with genes in the COVID-19 disease map (see Supplementary Material, Fig. S5), providing thus an independent support for the results presented here.

## Discussion

It is well-recognized that host genetics has an impact on COVID-19 progression and patient survival (30) as case-controls (31) genome-wide association studies (32–34) and whole-genome sequencing studies (35) have recently demonstrated. Since a large proportion of the >7000 reported rare diseases (over 80%) have a genetic etiology (3) it is likely that some of this genetic variation could also have an impact on COVID-19 prognosis. Actually, the interaction between rare diseases and other diseases has been studied to both, gain insights into disease’s mechanisms and evaluate the potential risk of vulnerable groups to other diseases (36–38). This is of particular relevance in the recent COVID pandemics, due to the potential cross-effects with other respiratory, hematologic chronic diseases or weakened immune system (39), not necessarily negative, such as the case of Hodgkin lymphoma remission after SARS-CoV-2 infection (10). Moreover, this knowledge is also relevant for the management and care of patients with limiting rare diseases, who are totally dependent on their caregivers or have special needs, and to whom the isolation measures can be mentally, and physically, devastating (40).

Studies to identify molecular relations between diseases have been previously made including the use of common genes (41), functional linkages (42) protein–protein (43) or metabolic (44) interactions or integration of different biological information types (45). However, this type of evidence, like proximity in a protein interaction network, co-expression, etc., suggests a possible common functional role but does not imply a causal relationship. Conversely, the use of mechanistic models conveys a notion of causality encoded in the relationships between the elements involved in the model (22,29,46–49). Mechanistic models have demonstrated their performance in predicting the effect of mutations in the context of complex diseases (27), the effect of perturbations (23) or drugs (29). In this work, a systematic prediction of mechanistic interactions between all rare diseases and COVID-19 has been carried out for the first time. This approach has allowed the identification of rare diseases both, with potential risk or protective effects in COVID-19 patients. Furthermore, the mechanistic detail of the model enabled an evaluation of the most affected COVID-19 hallmarks for any interacting rare disease, which can help to establish specific clinical measures of care and prevention.

From the results obtained, that describe only the strongest effects among the significant ones, it is obvious that the number of potential interactions between rare diseases and the disease mechanism of COVID-19 is beyond the expectations. Given the interest that all the potential interactions detected can have for the rare disease clinical community all the significant interactions found are available in Supplementary Material, Tables S2 and S3. Because of the magnitude of the results obtained an exhaustive comment of all the findings is out of the scope of this work. However, some general remarks on the main interactions, hallmarks affected and genes involved deserve to be made.

The results shown here reveal a large range of rare diseases, 702 out of the 3854 with known causal genes analyzed, in which the LoF of the corresponding genes potentially interacts with the molecular mechanism of SARS-CoV-2 infection and downstream consequences in the human signaling pathways, either only directly (445 diseases) or only indirectly (328) or both (71 of them). The general disease category with the highest potential effect over the COVID-19 disease map is rare developmental defect during embryogenesis with 168 potentially interacting diseases. Rare neurologic, immune or neoplastic diseases have also a remarkable potential of interaction (119, 74 and 70 diseases, respectively). Table 1 shows the number of rare diseases, clustered by rare disease type, that have been found to interact with the COVID-19 either directly or indirectly or both. More specifically, diseases that alter the immune system have an obvious mechanistic link to COVID-19 prognosis, although the signaling circuits likely involved in these interactions are starting to be known more recently. For example, *TFRC, NF-KB, MAPK3K7, LCK, FERMT3, DOCK2* and several subunits of Inhibitor of the nuclear factor kappa B kinase (*IKBK*) genes are related to signaling circuits of T-cell receptor, Toll-like and HIF-1 signaling pathways, which are activated after COVID-19 infection (29). All these signaling circuits are related to immune response to COVID-19 and to a wide range of immunodeficiencies (Supplementary Material, Tables S2 and S3). Some genes causal of neoplasia-related disorders or developmental disorders could be related to COVID-19 due to their simultaneous implication in immune and inflammatory processes, and also due to their ability to control more general processes, such as cell replication and apoptosis mechanisms, used by the virus for its replication. Remarkably, a recent study evaluated the role of cancer-related pathways in SARS-CoV-2 infection, identifying four major signaling pathways at the intersection of COVID-19 and cancer: cytokine, type I interferon (*IFN-I*), androgen receptor (AR) and immune checkpoint signaling, suggesting the repurposing of anticancer treatment for the treatment of COVID-19 (50).

Among the results, several rare disease genes were found with significant impact over COVID-19 signaling circuits related to viral infection, that trigger antiviral defense or the replication machinery, such as *IRF3*, *ALDH18A1, ALG8, COL4A, ABCC9, STAT3, DNAJB1*, *DNAJC3, RB1, PTEN, SLC2A* and *STAT3*, most of them are responsible of neoplasia or autoimmune diseases. Rare diseases in which these genes are affected have a high potential of interaction with COVID-19 infections, presenting exacerbated symptomatology. As an example, mutations in the latter gene (*STAT3*) causes a rare genetic lymphoproliferative syndrome characterized by early onset recurrent infections (human phenotype ontology—HPO—codes HP:0002716 and HP:0002719), and variable autoimmune disorders (HP:0002960), including hemolytic anemia (HP:0001903 and HP:0001890), thrombocytopenia (HP:0001973 and HP:0001873) and inflammatory lung disease [HP:0002783, HP:0006515 and HP:0006532; (51)], as well as decreased regulatory T-cells, hypogammaglobulinemia and reduction in memory B cells [HP:0001876; (52)]. Interestingly, recent studies have identified that the aberrant STAT pathway is a central mechanism of COVID-19 (13,53).

Moreover, connections of COVID-19 to hematologic diseases have been found, especially to those causing anemia, thrombocytopenia and other blood cell anomalies, caused by mutations or deficiencies in genes *WAS*, *FYB1*, *SRC*, among others (Supplementary Material, Tables S2 and S3), causing diseases such as X-linked severe congenital neutropenia (ORPHA:86788, HP:0001875 and HP:0002718), Congenital autosomal recessive small-platelet thrombocytopenia (ORPHA:566192) or Hereditary thrombocytopenia with early-onset myelofibrosis (ORPHA:480851), respectively. Indeed, thrombosis (HP:0002625, HP:0004831, HP:0004420 and HP:0005305) and coagulopathies (HP:0003256 and HP:0001928) are common complications of COVID-19 (54,55), and even cases of anemia after COVID-19 have been described (56). Of particular relevance is *PRF1* gene (Supplementary Material, Table S3), in which some mutations encode a shorter perforin protein, unable to carry out its role of cell destruction and immune system regulation, leading to the exaggerated immune response (HP:0002958, HP:0003256, HP:0030356, HP:0011112, HP:0011118, HP:0002958, HP:0030783 and HP:0004313) characteristic of familial hemophagocytic lymphohistiocytosis (57), condition that could aggravate the COVID-19 infection response, leading to the cytokine storm and to severe COVID-19 (58). Indeed, mutations in this gene also cause Fatal post-viral neurodegenerative disorder (ORPHA:391343), a rare neuroinflammatory (HP:0033429) disease triggered by viral infection (59). Therefore, patients of these two diseases must be handled with especial care due to an innate predisposition to some COVID-19 symptoms.

Beyond these examples that illustrate confirmed interactions already reported between rare diseases and COVID-19 infection hallmarks, the approach presented here has detected potential interactions in ~10% of the 7000 of the rare diseases described (2) to date (see Supplementary Material, Tables S1 and S2). Obviously, the prevalence, onsets and specific conditions of the patients can be very different in each rare disease, thus, further thorough, expert analysis of the findings presented here is needed in order to evaluate the validity of all the interactions between rare diseases and COVID-19 and eventually validate them.

Here, the use of innovative mechanistic modeling methodologies has demonstrated its discovery possibilities for detecting potential interactions of rare diseases with COVID-19 but this approach can also be used for the identification of potential comorbidities of rare diseases, with other diseases, either present or arising in the future. This holistic modeling approach systematically evaluates all rare diseases at once, allowing a rapid and comprehensive analysis of potential risks faced by rare diseases patients.

## Materials and Methods

### Data sources

Gene expression data from 948 non-diseased individuals across 30 tissue sites, comprising a total of 17 382 samples and ~20 000 gene expression measurements each, were downloaded from the GTEx Portal [(60); GTEx Analysis V8].

A list of 2518 disease genes, reported to be causative of a total of 3854 diseases, subclassified into 34 different types, was downloaded from Orphadata (version 1.3.6/4.1.72020-04-17) repository (http://www.orphadata.org/; see Supplementary Material, Table S1).

### Data preprocessing

Gene expression data were normalized using the trimmed mean of M values (TMM) method (61).

### Mechanistic model of the COVID-19 disease map

Here, we use the version of the COVID-19 disease map already implemented and publicly available in the CoV-Hipathia tool (14). Briefly, the intersection of the SARS-CoV-2 virus-human interactome (62) with Kyoto encyclopedia of genes and genomes (KEGG) pathways (63) is used to define regions within the whole set of KEGG pathways that account for the molecular mechanism of the viral infection and the downstream consequences. Such regions are decomposed into elementary signaling circuits (with one or more initial receptor nodes and one unique final effector node). Only signaling circuits triggering functions related with the COVID-19 hallmarks (i) Host-virus interaction, (ii) inflammatory response, (iii) immune activity, (iv) antiviral defense, (v) endocytosis, (vi) replication, (vii) energetics, (viii) adhesion and (ix) apoptosis according to Uniprot annotations (64), were considered (13,14). A total of 276 circuits were defined in the current version of the COVID-19 disease map (13,14).

The mechanistic model of the COVID-19 disease map allows transforming the transcriptional and/or mutational profiles of the genes involved in the map into signaling activity profiles of the corresponding signaling circuits (14,22). In particular, the hipathia (22,65) algorithm has been used here, which has demonstrated to outperform other similar algorithms (46). Specifically, the mechanistic model simulates the behavior of the signaling circuits of the COVID-19 disease map. Such circuits are described as directed graphs that connect receptor to effector proteins through a chain of activations and inhibitions carried out by intermediate proteins, which ultimately trigger specific functions in the cell (related to COVID-19 hallmarks). The mechanistic model simulates the transduction of the signal along the circuits considering the level of activity of the proteins that compose it. Here, as in many other modeling scenarios, gene expression values are taken as proxies of the presence of the corresponding activated proteins (66–71). Therefore, to be active, and consequently transmit the signal that ultimately triggers a specific function, a circuit requires the simultaneous presence of the whole chain of proteins that connect the receptor to the effector, as well the absence of inhibitor proteins that may compromise the transmission of the signal along the circuit. No matter of how the topology of the circuit is, for each node *n* within, the signal is propagated along the circuit nodes according to the following recursive rule:

(1) \begin{equation*} {S}_n={\upsilon}_n\cdot \left(1-\prod_{s_a\in A}\left(1-{s}_a\right)\right)\cdotp \prod_{s_i\in I}\left(1-{s}_i\right) \end{equation*}

where *S_n_* is the signal intensity for the current node *n*, *v_n_* is its normalized gene expression value, *A* is the set of activation signals (*s_a_*), arriving to the current node from activation edges, *I* is the set of inhibitory signals (*s_i_*) arriving to the node from inhibition edges. See (22) for details. This way of modeling signaling circuit activity conveys causality in a holistic context because the expression levels of the genes will determine the ultimate functional consequences, triggered by the effector proteins, following the activation/inhibition rules imposed by the relationships among the proteins in the signal circuit. Actually, changes in the activity of the nodes will be reflected (or remain unnoticed) in the last effector node, depending on the topology of each circuit.

The mechanistic model of the COVID-19 map can thus be used to transform gene expression profiles into COVID-19 hallmark activities (according to their corresponding signaling circuit activities).

### Inferring the impact of LoF mutations causative of rare diseases on the molecular mechanism of SARS-CoV-2 infection

Mechanistic models of the COVID-19 disease map can be used to assess which COVID-19 functionalities (COVID-19 hallmarks) triggered by the different signaling circuits are active in the disease in a given condition (14). Similarly, since mechanistic models convey the notion of causality, they can be used to specifically detect the effect of perturbations in genes of the COVID-19 disease map over the different COVID-19 hallmarks. Specifically, two scenarios are considered: (i) the disease gene with a LoF mutation is part of the COVID-19 disease map and (ii) the disease gene is outside the COVID-19 disease map.

### Predicted effect of rare disease genes integral to the COVID-19 disease map

Mechanistic models can be used to predict the effect that perturbations on genes belonging to the signaling circuits have over the resulting signaling activity of the disease map (29,72). Here, rare disease genes harboring LoF mutations are considered natural knock-downs and their effect on the disease map can be simulated by substituting their original expression level in a given condition by a near-zero value in the simulated condition (72). Therefore, normal gene expression data is used to define a set of normal controls, and for them another set is derived by simulating the corresponding LoF knock-downs, which is taken as the cases set. Thus, signaling activity profiles are inferred for the original controls and simulated condition cases and can be compared with detect circuits differentially activated as a consequence of the simulated knock-down.

In order to check the significance of the effect of the perturbation due to each knock-down of any of the diseases considered, a three steps procedure (27) is followed for each rare disease gene integral to the COVID-19 disease map: (i) the 17 382 samples corresponding to 30 tissues from the GTEx repository are taken as a control normal dataset, (ii) from this dataset a new (simulated) cases dataset is generated by simulating a knock-down of the gene corresponding to a specific rare disease, where the knock-down of a gene is simulated by substituting its expression level by a low value, while the rest of genes remain with the original expression levels [0.01; (27,72)], (iii) then, the mechanistic model is used to estimate the profiles of circuit activities in both, control and simulated disease datasets and (iv) the distribution of signaling values obtained for each circuit in the simulated disease dataset is compared with the corresponding distribution of signaling values obtained from the control dataset using the Kolmogorov–Smirnov statistic on two samples and the associated *P*-value for a two sided test (the null hypothesis is that both sets of samples are drawn from the same distribution). *P*-values are corrected for multiple testing by FDR (73). A disease gene is considered to have a significant effect on a circuit(s) if the null hypothesis can be rejected (α = 0.05).

Furthermore, the log-fold-change of the signal activity between the control and simulated populations can be further used to search for potential clusters along the diseases (using their linked genes) and the circuits.

### Potential effect of rare disease genes external to the COVID-19 disease map

In the case of genes that are not part of the COVID-19 disease map the effect of a knock-down cannot be directly inferred by the mechanistic model. For this case, a correlation-based test has been implemented as follows: let *g^o^* be a gene which is not part of the COVID-19 disease map, *c* a signaling circuit and *g^c^* the gene which has the highest absolute correlation with the circuit *c* among the genes that belong to *c*. We aim to infer if *g^o^* could be potentially associated with the circuit *c* by comparing the absolute correlation between *g^o^* and the circuit *c*, denoted by *r(g^o^, c)* and the absolute correlation between *g^c^* and *c*, denoted by *r(g^c^, c)*. The comparison between both correlations is carried out by means of the Hittner one-tailed test of significance for overlapping correlations (as the circuit activity links both) based on dependent groups [both came from the same samples; (74)], as implemented in the *cocor* R package (75). Thus, the analysis tests if *r(g^o^, c)* is greater than *r(g^c^, c)* by taking into account the intercorrelation between the genes *g^o^* and *g^c^*, *r(g^o^, g^c^)*. Finally, the described procedure is carried out for each gene which does not belong to the COVID-19 disease map and each circuit that makes it up, the *P*-values obtained are corrected for multiple testing [FDR; (73)] and a disease gene is considered to have a potential influence on the circuit if the associated *P*-values is significant (alpha = 0.05).

The high stringency of the procedure aims to retain only those disease genes with a correlation with the circuit activity superior to those shown by the genes belonging to the circuit, which is more likely to be due to a causal relationship. Note that in all cases absolute correlations were used.

### Potential biological connections that justify the interaction between external genes and the COVID-19 disease map

Since a significant correlation does not imply a causal relationship we have used two methods to assess whether there is a biological link that supports the correlation or not.

First, a protein–protein interaction analysis of the disease genes and the circuits of the disease map based on the combined score provided by The Search Tool for the Retrieval of Interacting Genes/Proteins [(STRING); (http://string-db.org); (76)] was conducted. The R package *stringdb* (v 2.4.0) was used, considering the following evidence thresholds suggested by STRING (76): no confidence (0–0.15), low confidence (0.15–0.4), medium confidence (0.4–0.7), high confidence (0.7–0.9) and highest confidence (>0.9; see https://version11.string-db.org/help/getting_started/for an updated threshold list). The combined interaction score between a disease gene *g* and any given circuit *c* was defined as the maximum of the combined interaction scores between *g* and the genes that compose *c*.

In addition, the ToppGene tool (77) for gene list enrichment in functional connections, an annotation-based gene prioritization system based a fuzzy-based similarity measure to compute the similarity between any two genes based on semantic annotations, was used to confirm or discard functional connections between significantly correlated genes and signaling circuit activities in the COVID-19 disease map.

## Supplementary Material

Additional_Figure_1_ddac007

Additional_Figure_2_ddac007

Additional_Figure_3_ddac007

Additional_Figure_4_ddac007

Additional_Figure_5_ddac007

Additional_table_1_ddac007

Additional_table_2_ddac007

Additional_Table_3_ddac007
