# Supplementary figures and images for: Discovering potential interactions between rare diseases and COVID-19 by combining mechanistic models of viral infection with statistical modeling
Source: Hum Mol Genet. 2022 Jan 12;31(12):2078–89. doi: 10.1093/hmg/ddac007 (PMC9239744; doi:10.1093/hmg/ddac007)

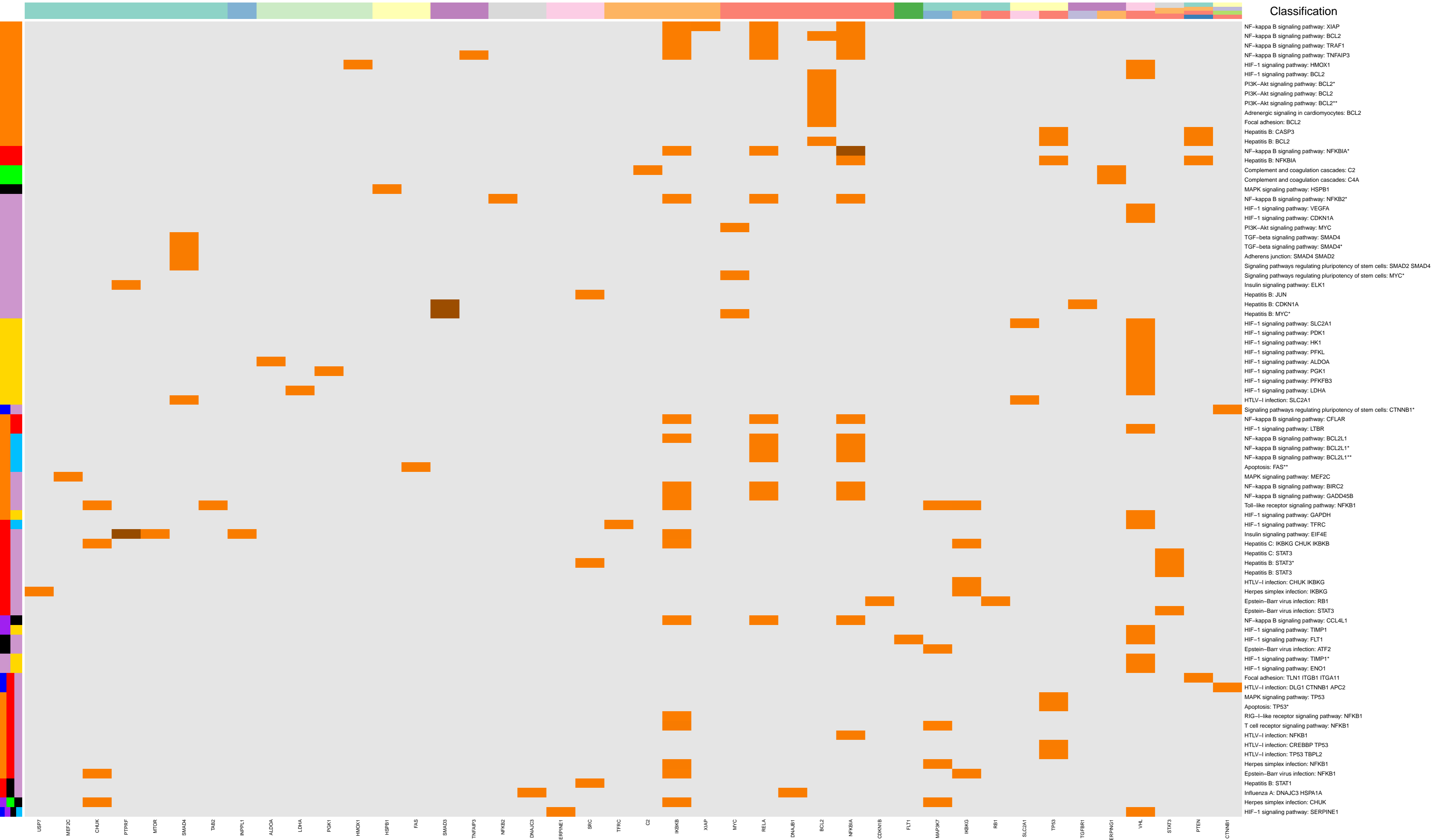

Hallmark

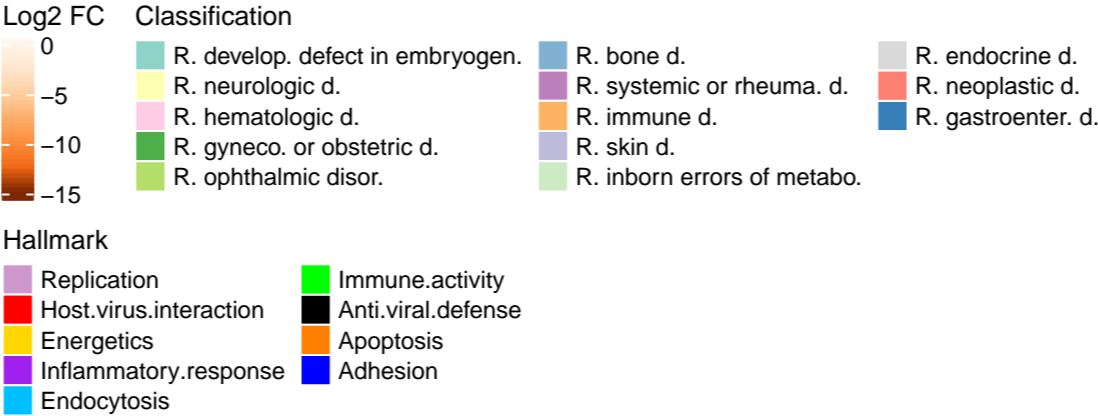

Supplement: Additional_Figure_1_ddac007 [file additional_figure_1_ddac007.pdf]

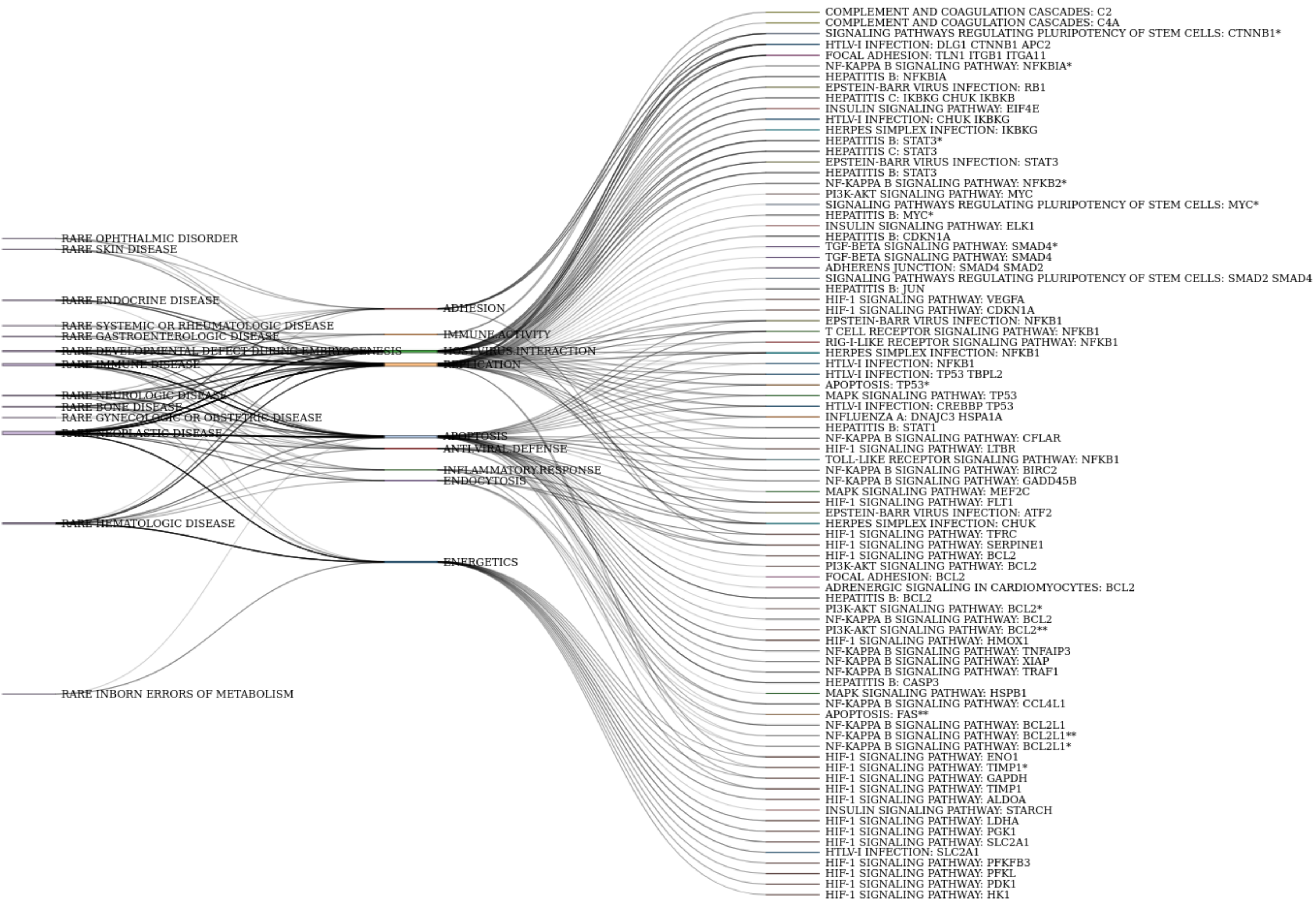

Supplement: Additional_Figure_2_ddac007 [file additional_figure_2_ddac007.pdf]

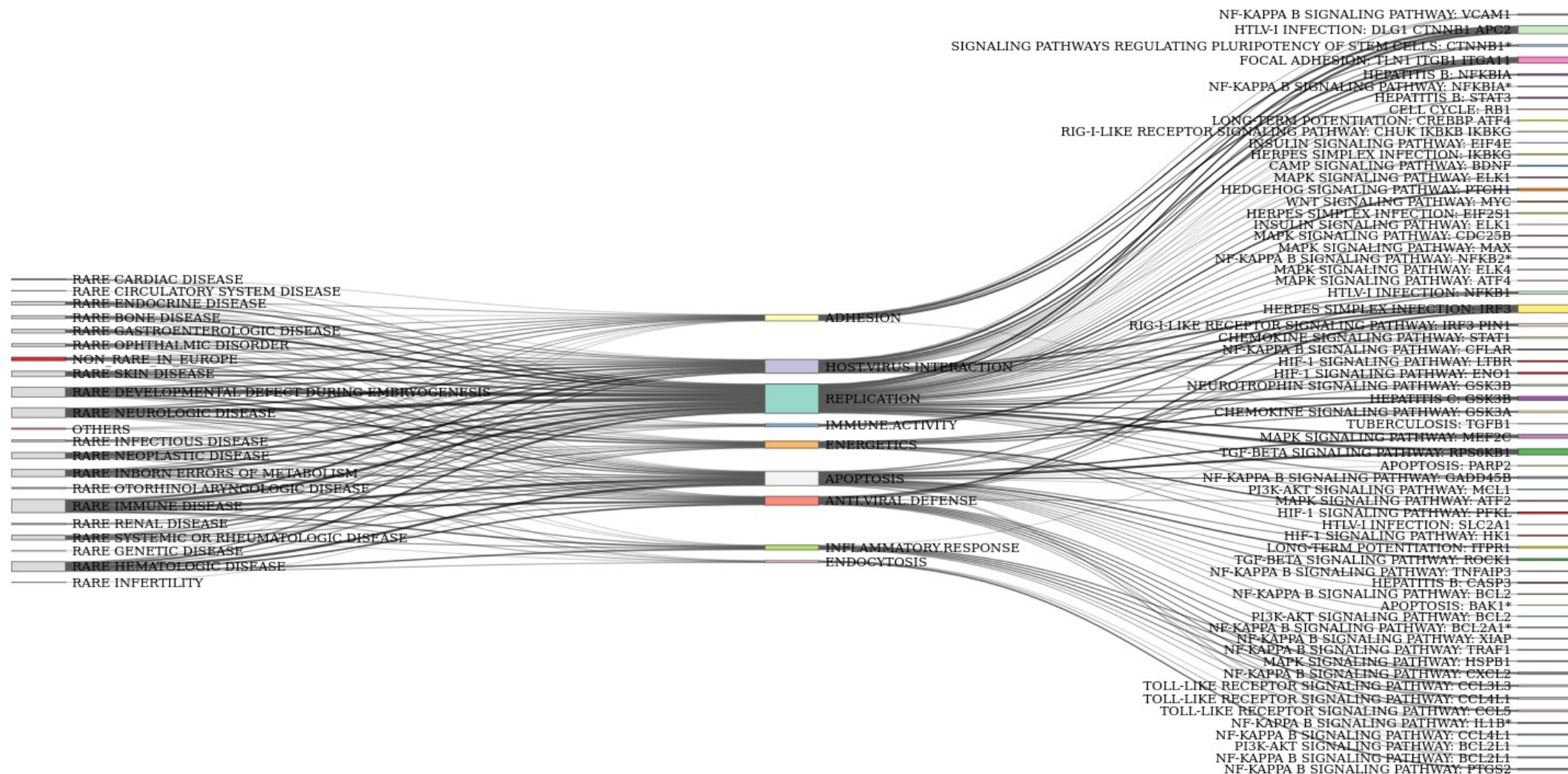

Supplement: Additional_Figure_4_ddac007 [file additional_figure_4_ddac007.pdf]
