# Supplementary material for: Discovering potential interactions between rare diseases and COVID-19 by combining mechanistic models of viral infection with statistical modeling
Source: Hum Mol Genet. 2022 Jan 12;31(12):2078–89. doi: 10.1093/hmg/ddac007 (PMC9239744; doi:10.1093/hmg/ddac007)
Supplement: Additional_Figure_3_ddac007 [file additional_figure_3_ddac007.pdf]

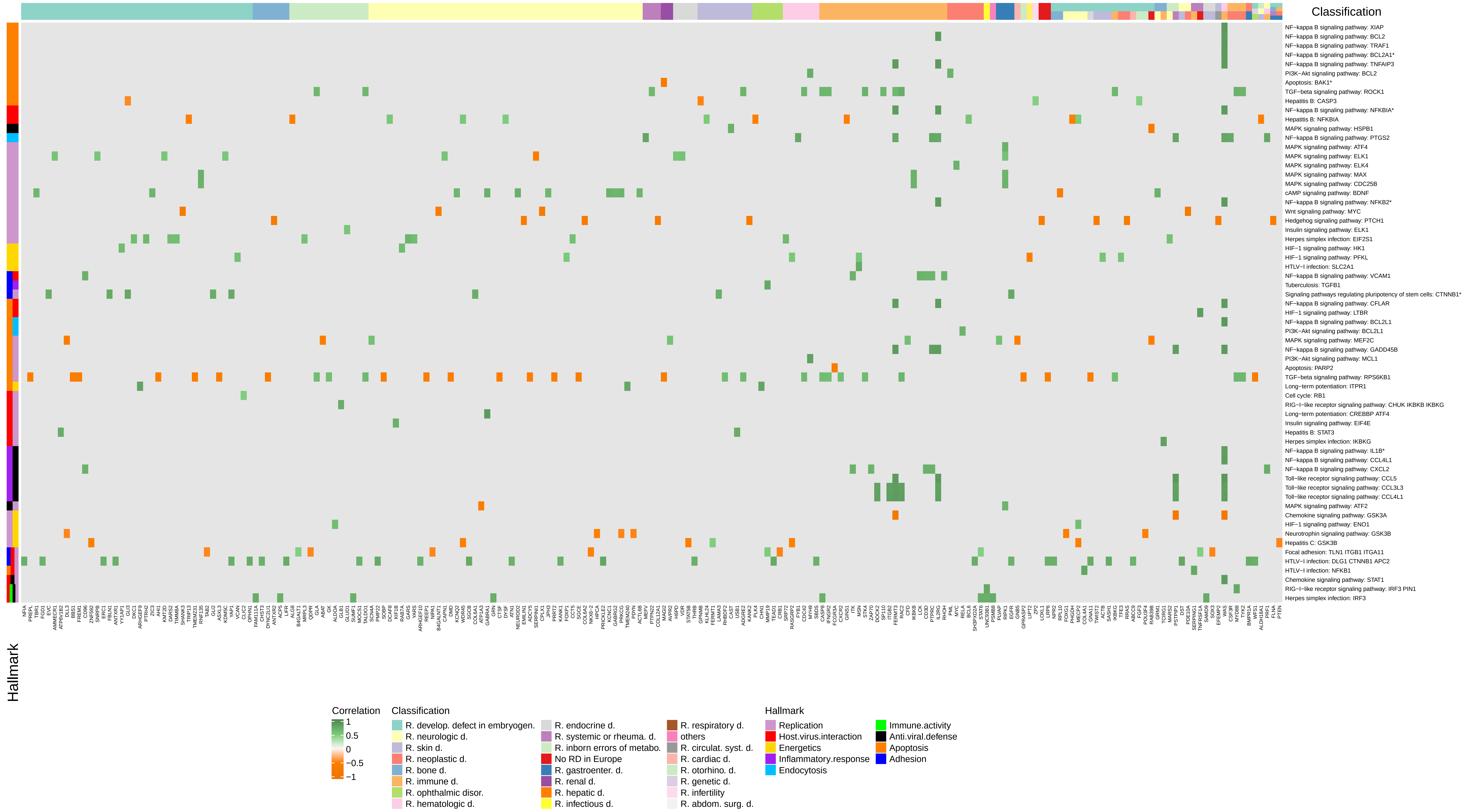
Classification
NF−kappa B signaling pathway: XIAP
NF−kappa B signaling pathway: BCL2
NF−kappa B signaling pathway: TRAF1
NF−kappa B signaling pathway: BCL2A1*
NF−kappa B signaling pathway: TNFAIP3
PI3K−Akt signaling pathway: BCL2
Apoptosis: BAK1*
TGF−beta signaling pathway: ROCK1
Hepatitis B: CASP3
NF−kappa B signaling pathway: NFKBIA*
Hepatitis B: NFKBIA
MAPK signaling pathway: HSPB1
NF−kappa B signaling pathway: PTGS2
MAPK signaling pathway: ATF4
MAPK signaling pathway: ELK1
MAPK signaling pathway: ELK4
MAPK signaling pathway: MAX
MAPK signaling pathway: CDC25B
cAMP signaling pathway: BDNF
NF−kappa B signaling pathway: NFKB2*
Wnt signaling pathway: MYC
Hedgehog signaling pathway: PTCH1
Insulin signaling pathway: ELK1
Herpes simplex infection: EIF2S1
HIF−1 signaling pathway: HK1
HIF−1 signaling pathway: PFKL
HTLV−I infection: SLC2A1
NF−kappa B signaling pathway: VCAM1
Tuberculosis: TGFB1
Signaling pathways regulating pluripotency of stem cells: CTNNB1*
NF−kappa B signaling pathway: CFLAR
HIF−1 signaling pathway: LTBR
NF−kappa B signaling pathway: BCL2L1
PI3K−Akt signaling pathway: BCL2L1
MAPK signaling pathway: MEF2C
NF−kappa B signaling pathway: GADD45B
PI3K−Akt signaling pathway: MCL1
Apoptosis: PARP2
TGF−beta signaling pathway: RPS6KB1
Long−term potentiation: ITPR1
Cell cycle: RB1
RIG−I−like receptor signaling pathway: CHUK IKBKB IKBKG
Long−term potentiation: CREBBP ATF4
Insulin signaling pathway: EIF4E
Hepatitis B: STAT3
Herpes simplex infection: IKBKG
NF−kappa B signaling pathway: IL1B*
NF−kappa B signaling pathway: CCL4L1
NF−kappa B signaling pathway: CXCL2
Toll−like receptor signaling pathway: CCL5
Toll−like receptor signaling pathway: CCL3L3
Toll−like receptor signaling pathway: CCL4L1
MAPK signaling pathway: ATF2
Chemokine signaling pathway: GSK3A
HIF−1 signaling pathway: ENO1
Neurotrophin signaling pathway: GSK3B
Hepatitis C: GSK3B
Focal adhesion: TLN1 ITGB1 ITGA11
HTLV−I infection: DLG1 CTNNB1 APC2
HTLV−I infection: NFKB1
Chemokine signaling pathway: STAT1
RIG−I−like receptor signaling pathway: IRF3 PIN1
Herpes simplex infection: IRF3
Hallmark
Correlation
1
0.5
0
−0.5
−1
Classification
R. develop. defect in embryogen.
R. neurologic d.
R. skin d.
R. neoplastic d.
R. bone d.
R. immune d.
R. ophthalmic disor.
R. hematologic d.
R. endocrine d.
R. systemic or rheuma. d.
R. inborn errors of metabo.
No RD in Europe
R. gastroenter. d.
R. renal d.
R. hepatic d.
R. infectious d.
R. respiratory d.
others
R. circulat. syst. d.
R. cardiac d.
R. otorhino. d.
R. genetic d.
R. infertility
R. abdom. surg. d.
Hallmark
Replication
Host.virus.interaction
Energetics
Inflammatory.response
Endocytosis
Immune.activity
Anti.viral.defense
Apoptosis
Adhesion
