# Supplementary material for: Discovering potential interactions between rare diseases and COVID-19 by combining mechanistic models of viral infection with statistical modeling
Source: Hum Mol Genet. 2022 Jan 12;31(12):2078–89. doi: 10.1093/hmg/ddac007 (PMC9239744; doi:10.1093/hmg/ddac007)
Supplement: Additional_Figure_5_ddac007 [file additional_figure_5_ddac007.pdf]

Results

Go To Start Page

Training Parameters [Show All]

Training Gene Subnetwork [Hide All] Display pValues and Scores as Scientific (4 significant digits)

Number of genes: 15135
Number of interactions: 518231
Too many interactions to display

Format: XGMML (An XML based format compatible with Cytoscape) Export

Test Genes [Hide All]

Rank	ID	Name	Interactant count	Score
1	1675	CFD	1348	1.032E-3
2	10413	YAP1	674	4.974E-4
3	5371	PML	300	2.653E-4
4	85358	SHANK3	269	2.533E-4
5	6134	RPL10	456	2.528E-4
6	81622	UNC93B1	371	2.516E-4
7	7030	TFE3	323	2.442E-4
8	2896	GRN	202	2.394E-4
9	22948	CCT5	300	2.246E-4
10	7879	RAB7A	298	2.164E-4
11	657	BMPR1A	139	1.879E-4
12	604	BCL6	166	1.696E-4
13	1462	VCAN	32	1.538E-4
14	25959	KANK2	236	1.502E-4
15	4478	MSN	170	1.491E-4
16	2767	GNA11	71	1.434E-4
17	2192	FBLN1	98	1.415E-4
18	9319	TRIP13	205	1.399E-4
19	51651	PTRH2	161	1.387E-4
20	6628	SNRPB	226	1.347E-4
21	26227	PHGDH	205	1.322E-4
22	114928	GPRASP2	98	1.273E-4
23	1736	DKC1	213	1.125E-4

Rank	ID	Name	Interactant count	Score
24	1822	ATN1	138	1.080E-4
25	30008	EFEMP2	132	1.041E-4
26	4204	MECP2	180	1.041E-4
27	1756	DMD	106	9.310E-5
28	7454	WAS	82	9.048E-5
29	23095	KIF1B	116	8.936E-5
30	9644	SH3PXD2A	102	8.897E-5
31	2617	GARS1	154	8.757E-5
32	79651	RHBDF2	116	8.425E-5
33	667	DST	118	8.272E-5
34	3431	SP110	113	8.018E-5
35	2746	GLUD1	131	7.979E-5
36	6789	STK4	92	7.720E-5
37	23085	ERC1	96	6.983E-5
38	5832	ALDH18A1	103	6.884E-5
39	5376	PMP22	63	6.761E-5
40	7068	THRB	82	6.579E-5
41	6888	TALDO1	92	6.559E-5
42	166336	PRICKLE2	57	6.348E-5
43	84168	ANTXR1	62	6.318E-5
44	9051	PSTPIP1	95	6.242E-5
45	6731	SRP72	104	6.183E-5
46	2290	FOXG1	93	6.179E-5
47	2710	GK	67	5.748E-5
48	2683	B4GALT1	65	5.590E-5
49	2720	GLB1	73	5.561E-5
50	11117	EMILIN1	67	5.493E-5
51	8291	DYSF	55	5.389E-5
52	7003	TEAD1	51	5.385E-5
53	10733	PLK4	80	5.383E-5
54	478	ATP1A3	57	5.277E-5
55	8085	KMT2D	65	5.236E-5
56	51119	SBDS	74	5.227E-5
57	8242	KDM5C	73	5.196E-5
58	11222	MRPL3	128	5.140E-5
59	50717	DCAF8	76	5.107E-5
60	54806	AHI1	67	4.967E-5
61	83706	FERMT3	61	4.950E-5
62	8565	YARS1	73	4.949E-5
63	5696	PSMB8	81	4.797E-5
64	79583	TMEM231	41	4.773E-5
65	1794	DOCK2	62	4.710E-5

Rank	ID	Name	Interactant count	Score
66	27086	FOXP1	59	4.619E-5
67	831	CAST	58	4.358E-5
68	55157	DARS2	70	4.327E-5
69	26191	PTPN22	45	4.246E-5
70	9863	MAGI2	38	4.171E-5
71	285362	SUMF1	32	3.947E-5
72	7466	WFS1	47	3.901E-5
73	23328	SASH1	39	3.901E-5
74	399	RHOH	32	3.829E-5
75	2533	FYB1	26	3.765E-5
76	2214	FCGR3A	26	3.763E-5
77	1123	CHN1	29	3.657E-5
78	118429	ANTXR2	15	3.608E-5
79	9639	ARHGEF10	45	3.571E-5
80	2566	GABRG2	19	3.571E-5
81	79053	ALG8	39	3.460E-5
82	6443	SGCB	32	3.424E-5
83	4774	NFIA	54	3.198E-5
84	9581	PREPL	63	3.040E-5
85	582	BBS1	42	3.036E-5
86	1678	TIMM8A	48	3.029E-5
87	10846	PDE10A	22	2.988E-5
88	10815	CPLX1	44	2.985E-5
89	18	ABAT	47	2.785E-5
90	54	ACP5	28	2.607E-5
91	54800	KLHL24	26	2.573E-5
92	2554	GABRA1	26	2.412E-5
93	9640	ZNF592	38	2.398E-5
94	55249	YY1AP1	30	2.387E-5
95	51412	ACTL6B	32	2.273E-5
96	3785	KCNQ2	23	2.192E-5
97	65055	REEP1	27	2.143E-5
98	5860	QDPR	31	2.113E-5
99	11152	WDR45	30	2.084E-5
100	554	AVPR2	17	2.078E-5
101	23189	KANK1	25	2.046E-5
102	55612	FERMT1	24	1.980E-5
103	6329	SCN4A	15	1.814E-5
104	3208	HPCA	23	1.799E-5
105	116442	RAB39B	27	1.774E-5
106	2717	GLA	25	1.755E-5
107	57338	JPH3	10	1.735E-5

Rank	ID	Name	Interactant count	Score
108	23229	ARHGEF9	23	1.705E-5
109	30817	ADGRE2	20	1.694E-5
110	4983	OPHN1	20	1.630E-5
111	83483	PLVAP	12	1.605E-5
112	79650	USB1	31	1.566E-5
113	4882	NPR2	14	1.537E-5
114	1303	COL12A1	11	1.462E-5
115	84282	RNF135	20	1.459E-5
116	9949	AMMECR1	23	1.413E-5
117	117581	TWIST2	12	1.407E-5
118	51626	DYNC2LI1	19	1.369E-5
119	4210	MEFV	18	1.361E-5
120	3746	KCNC1	13	1.337E-5
121	10225	CD96	15	1.316E-5
122	112476	PRRT2	17	1.288E-5
123	10716	TBR1	17	1.188E-5
124	9837	GINS1	15	1.168E-5
125	6658	SOX3	16	1.155E-5
126	92935	MARS2	16	1.101E-5
127	5551	PRF1	10	1.090E-5
128	10457	GPNMB	12	8.967E-6
129	4337	MOCS1	14	8.769E-6
130	54809	SAMD9	13	7.903E-6
131	63901	FAM111A	11	7.384E-6
132	7547	ZIC3	8	7.295E-6
133	5173	PDYN	10	7.027E-6
134	123606	NIPA1	3	6.835E-6
135	10060	ABCC9	9	6.805E-6
136	9469	CHST3	7	6.537E-6
137	1193	CLIC2	8	6.210E-6
138	4016	LOXL1	8	5.908E-6
139	80816	ASXL3	9	5.529E-6
140	2583	B4GALNT1	7	5.526E-6
141	23418	CRB1	8	5.496E-6
142	9563	H6PD	7	5.036E-6
143	5274	SERPINI1	4	4.963E-6
144	55084	SOBP	7	4.516E-6
145	8910	SGCE	6	4.370E-6
146	84504	NKX6-2	3	4.197E-6
147	4761	NEUROD2	4	3.163E-6
148	2121	EVC	5	3.123E-6
149	5456	POU3F4	5	3.021E-6

Rank	ID	Name	Interactant count	Score
150	387787	LIPT2	4	2.538E-6
151	4327	MMP19	1	1.620E-6
152	158326	FREM1	2	1.427E-6
153	7783	ZP2	2	1.424E-6
154	339453	TMEM240	1	6.539E-7
155	10683	DLL3	1	5.863E-7
